# Medical students’ perception of lesbian, gay, bisexual, and transgender (LGBT) discrimination in their learning environment and their self-reported comfort level for caring for LGBT patients: a survey study

**DOI:** 10.1080/10872981.2017.1368850

**Published:** 2017-08-30

**Authors:** Nassr Nama, Paul MacPherson, Margaret Sampson, Hugh J. McMillan

**Affiliations:** ^a^ Department of Pediatrics, University of British Columbia, Vancouver, Canada; ^b^ Faculty of Medicine, University of Ottawa, Ottawa, Canada; ^c^ Division of Infectious Diseases, University of Ottawa, Ottawa, Canada; ^d^ Department of Volunteers, Communication and Information Resources, Children’s Hospital of Eastern Ontario, Ottawa, Canada; ^e^ Department of Pediatrics, University of Ottawa, Ottawa, Canada

**Keywords:** Undergraduate medical education, LGBT (lesbian, gay, bisexual, or transgender) persons, social discrimination, cultural competence, healthcare disparities

## Abstract

**Background**: Historically, medical students who are lesbian, gay, bisexual or transgendered (LGBT) report higher rates of social stress, depression, and anxiety, while LGBT patients have reported discrimination and poorer access to healthcare.

**Objective**: The objectives of this study were: (1) to assess if medical students have perceived discrimination in their learning environment and; (2) to determine self-reported comfort level for caring for LGBT patients.

**Design**: Medical students at the University of Ottawa (N = 671) were contacted via email and invited to complete a confidential web-based survey.

**Results**: Response rate was 15.4% (103/671). This included 66 cis-gender heterosexuals (64.1%) and 37 LGBT students (35.9%). Anti-LGBT discrimination had been witnessed by 14.6% and heterosexism by 31.1% of respondents. Anti-LGBT discrimination most often originated from fellow medical students. Respondents who self-identified as LGBT were more likely to have perceived heterosexism (favoring opposite-sex relationships) (OR = 8.2, p < 0.001) or anti-LGBT discrimination (OR = 6.6, p = 0.002). While half of LGBT students shared their status with all classmates (51.4%), they were more likely to conceal this from staff physicians (OR = 27.2, p = 0.002). Almost half of medical students (41.7%) reported anti-LGBT jokes, rumors, and/or bullying by fellow medical students and/or other members of the healthcare team. Still, most respondents indicated that they felt comfortable with and capable of providing medical care to LGBT patients (≥83.5%), and were interested in further education around LGBT health issues (84.5%).

**Conclusion**: Anti-LGBT discrimination and heterosexism are noted by medical students, indicating a suboptimal learning environment for LGBT students. Nonetheless, students report a high level of comfort and confidence providing health care to LGBT patients.

## Introduction

One of the goals of undergraduate medical education is to ensure that graduates can provide medical care to a wide range of patient populations. It is also important that the learning environment be inclusive and welcoming towards those that may belong to visible and non-visible minorities, not only to ensure that members of such minority groups succeed but also to model an atmosphere of respect and inclusivity towards all members of the society.

Despite this, disparities in health based on sexual orientation and gender identity are well documented and researched [1,2]. This inequality is evident in terms of access to the healthcare system and use of preventive health services [3,4]. As such, patients who identify as lesbian, gay, bisexual or transgender (LGBT) are at an increased risk for cancer, mental health disorders, substance abuse, and sexually transmitted infections [2,5,6]. Data shows that LGBT individuals receive poorer healthcare and often report a considerable degree of discrimination by medical students and practicing physicians. A recent survey indicated that transgender individuals may avoid seeking medical care because of their trans-status [7].

Fortunately, there has been a societal shift towards greater acceptance of LGBT issues [8]. This has been accompanied by a significant decline in explicit bias (discrimination, hate speech) and negative attitudes towards sexual and gender minorities [9]. With shifts in society’s attitude towards minorities, explicit bias becomes socially unacceptable, while implicit bias remains widespread [10,11]. Implicit bias refers to individuals’ perception and stereotypes without conscious intention. The prevalence of such a bias in the healthcare setting constitutes an unaddressed part of the medical curriculum [10,12], and various degrees of homophobia and discrimination continue to be noted by both physicians and trainees [13–15].

Medical students are often exposed to this bias early on in their training. As such, an overwhelming majority of medical school applicants did not want to disclose their orientation, and some feared that it would result in rejection [15,16]. Moreover, a significant portion of medical students described the climate at their institution as ‘non-inclusive’ [13,15,17]. This plays a major role in students concealing their identity or sexual orientation in the work place. Some students have even been deterred from participating in professional activities related to LGBT health issues, as they were poorly recognized, and that visibility might be associated with professional risks [18].

In general, concealment of sexual identity and/or orientation has been linked to an increase stress level and a negative effect on health outcomes [19–21]. This is also true for medical trainees, and can affect the academic success of LGBT students [22,23]. Studies show a correlation between depression in LGBT medical students and the non-inclusivity of campus climate as well as the discomfort of disclosure of sexual orientation [15]. This suggests that targeting these factors has the potential to counter the increased risk for mental health disorders among LGBT students.

As societal views of sexual orientation and gender minorities vary significantly among cultures and populations, it is important to assess whether these disparities are present in the Canadian medical education system. In this study, we aimed to: (1) determine medical studentsʼ comfort level and knowledge working with colleagues and caring for patients who identify as LGBT; and (2) determine if medical students have witnessed or perceived any bias against LGBT persons from fellow medical students, residents, allied health staff (e.g. nurses, physiotherapists) and/or staff physicians.

## Methods

This study was a questionnaire-based survey that targeted all medical students of the University of Ottawa. Six-hundred-seventy-one students from all four years were invited to complete a confidential and anonymous questionnaire (Online, Appendix 1). Undergraduate level students were targeted as they have a more defined and structured curriculum where we could influence change. An original email containing a description of the study and the link for the survey was sent in February 2017 by a representative of the undergraduate medical education (UGME) office. The undergraduate medical program at the University is Ottawa has two streams, English and French. Emails and survey links were sent in both languages. To maximize our response rate, we sent out the survey during a period that was optimal for students from all four years. We avoided conflict with examinations or vacations. Two reminder e-mails were also sent out two weeks apart. Study participation was completely voluntary, and no compensation was offered to those who completed the survey. The study was approved by the Children’s Hospital of Eastern Ontario (CHEO) Research Ethics Board (REB) (#16-46X) and the Ottawa Health Science Network (OHSN) REB (#20160835-01H). No identifying information was used at any point in this study. Consent was deemed implied, due to the voluntary nature of participation.

### Study survey

The survey was based on a previous survey conducted at one of the teaching hospitals, which served as the pilot [24]. The survey was conducted using RedCap [25]. Students were divided in two groups, LGBT and cis-gender heterosexuals, defined as individuals whose gender identity matches their birth sex and that are heterosexual. The survey assessed whether students have witnessed or perceived any heterosexism or bias against LGBT individuals. Heterosexism was defined in the survey as favoring opposite-sex sexuality and relationships and/or portraying opposite-sex relationships as the only norm and therefore superior. Students were asked to score their own comfort level defining LGBT related terms, among which was two spirited, a term used by North American Indigenous peoples to refer to individuals that house both male and female spirits [26]. Finally, students were also asked to rate their agreement with multiple statements on a five-point Likert rating scale (1 being strongly agree and 5 being strongly disagree).

### Statistical analysis

Data were exported from RedCap and analyzed using SAS (version 9.4; SAS Institute, Cary, USA). Figures were generated using GraphPad Prism (version 7.0b; GraphPad Software, Inc., La Jolla, USA). Fisher’s exact test was used to compare respondents’ characteristics and responses between LGBT and non-LGBT students. For odds ratio calculation, when one of the cells was zero, 0.5 was added to all four cells [27]. Odds ratio was reported as per Cole [28]. For ordinal data on Likert scale, median and interquartile range (IQR) were used. Because data were non-continuous and not normally distributed, nonparametric tests were performed (Wilcoxon-Mann-Whitney’s). Wilcoxon signed-rank t-test was used to compare attitudes of students towards LGB versus transgender issues.

A minimum cell size of five respondents was required to report any subgroup analysis based upon a report that reasoned publication of small data subsets could, in theory, pose a threat to confidentiality [29].

## Results

### Survey respondents

Responses were obtained from 123 students. Of these 103 were complete submissions and used in the analysis, representing a response rate of 15.4% (103/671). Female-identifying students outweighed male-identifying ones (58.3% vs. 40.8%), in accordance with the overall gender ratio of medical students in the program (female 54% and male 46%, p = 0.39) (Table 1). A decrease in response rate between different years of study was noted; however, not statistically significant. The majority of study respondents identified as heterosexuals (63.1%, n = 65). This was followed by gay (18.4%, n = 19) and lesbian (7.8%, n = 8). A total of 14 students (13.6%) identified as being of other sexual orientations, including but not limited to bisexual, queer, or questioning. The number of students in each of these groups was lower than the minimum 5 set by our protocol to preserve confidentiality, and as such they were not reported separately. Overall, sixty-six students identified as cis-gender heterosexual (64.1%), and the remaining thirty-seven (35.9%) are referred to as LGBT students in this analysis. Comparison between LGBT students and non-LGBT students showed no statistical differences in regards to the year of study (p = 0.37) or prior education (p = 0.70).

**Table 1.** Characteristics of survey respondents (N = 103).

	Number	Percentage (%)
**Year of study**		
Year 1	34	33.0
Year 2	24	23.3
Year 3	27	26.2
Year 4	15	14.6
Prefer not to answer	3	2.9
**Education prior to start of medical training**		
Started Bachelor degree	10	9.7
Completed Bachelor degree	77	74.8
Completed Master degree	15	14.6
Prefer not to answer	1	1.0
**Gender**		
Female	60	58.3
Male	42	40.8
Other	1	1.0
**Sexual orientation^a^**		
Heterosexual	65	63.1
Gay	19	18.4
Lesbian	8	7.8
Other^b^	14	13.6
Prefer not to answer	2	1.9

^a^Numbers do not add up to total number of surveys as respondents were allowed to select multiple choices.

^b^Other includes: Queer, questioning, bisexual or other. Data were not presented separately as a minimum cell size of five respondents was required to guarantee confidentiality.

### Disclosure of sexual orientation and/or gender identity

**Figure 1.**
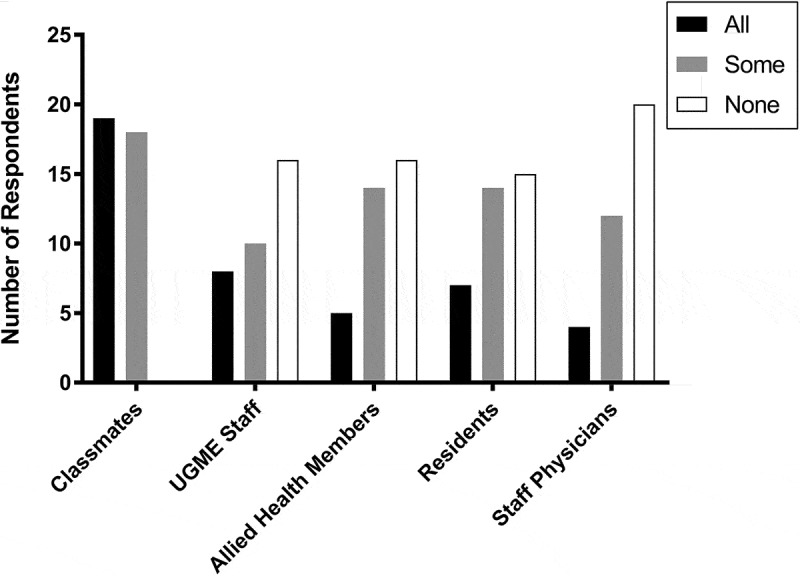
Disclosure of sexual orientation and/or gender identity. LGBT identifying students were asked to whom of their colleagues and other members of the healthcare team they have disclosed their status. Allied health members: Nurses, physiotherapists, occupational therapists, etc. UGME: Undergraduate Medical Education office.

None of the LGBT students (N = 37) who completed the survey concealed their identity from all their classmates (Figure 1). Slightly more than half of respondents (51.4%, n = 19/37) disclosed their status to all their classmates, and the remaining were out to some (48.6%, n = 18). Students had higher odds of disclosing to some or all the staff members at the UGME office, allied health members (i.e. nurses, physiotherapists, occupational therapists, etc.) and/or residents compared to staff physicians (OR = 27.2, 95%CI:1.44–517, p = 0.002). Most common reasons for concealment were a personal belief that this information is not anyone else’s business (48.6%, n = 18/37), concern for being stereotyped (45.9%, n = 17/37), and concern for being discriminated against (i.e., lost career opportunities) (37.8%, n = 14/37) (Online, Appendix 2).

### Discrimination

Almost a third of medical students (31.1%, n = 32) had witnessed heterosexism (Table 2). Fellow medical students were the most common source of heterosexism (87.5%, n = 28/32). Exposure to LGBT discrimination was less common than heterosexism. Only fifteen students (14.6%) said that they have witnessed discrimination against LGBT individuals. Medical students were also the most common source of such discrimination (73.3%, n = 11/15). LGBT identifying students had higher odds of witnessing or being exposed to heterosexism (OR = 8.2, 95%CI:2.93–23.5, p < 0.001), and anti-LGBT discrimination (OR = 6.6, 95%CI:1.71–30.3, p = 0.002). Among those who have witnessed anti-LGBT discrimination, 13 students indicated that these incidents might have led the victims to have a lesser sense of self-worth (86.7%, n = 13/15), and three students felt that the victim could have perceived the situation as a potential physical threat (20.0%, n = 3/15).

**Table 2.** Students’ exposure to anti-LGBT discrimination and heterosexism.

	Discrimination		Heterosexism	
Source	Number	Percentage (%)	Number	Percentage (%)
**None experienced**	88	85.4	71	68.9
**Yes**	15	14.6	32	31.1
Medical students	11	73.3	28	87.5
Staff physicians	5	33.3	18	56.3
Residents	3	20.0	10	31.3
OR staff	3	20.0	10	31.3
Nursing staff	2	13.3	7	21.9
UGME staff	1	6.7	6	18.8
Paramedics	0	0	1	3.1
PT /OT staff	0	0	0	0

LGBT: Lesbian, gay, bisexual, or transgender individuals. OR: Operation room. UGME: Undergraduate Medical Education office. PT: Physiotherapists. OT: Occupational therapists.

**Figure 2.**
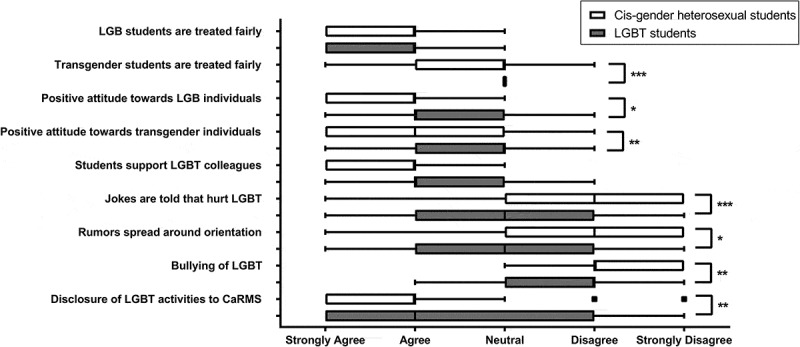
Medical students’ opinion on LGBT discrimination. Box and whiskers based on Tukey’s method. Box delineates interquartile range (IQR), with the vertical line inside the box showing the median. Whiskers extend from the lowest value or the 25th percentile minus 1.5 IQR, whichever is lower, to the highest value or the 75th percentile plus 1.5 IQR, whichever is higher. Data points that are outside of the whiskers’ range are plotted independently. Data are presented separately for LGBT-identifying and cis-gender heterosexual students. Comparison between the two groups is performed using Wilcoxon-Mann-Whitney’s test. All significant differences are noted on the graph. * p < 0.05, ** p < 0.01, *** p < 0.001. CaRMS: Canadian Residency Matching Service. LGBT: Lesbian, Gay, Bisexual and Transgender individuals.

Overall, students agreed that lesbian, gay, and bisexual (LGB) students are treated fairly in the program (median = 2, IQR:1–2), while the students’ opinion was mitigated in regards to treatment of transgender students (median = 3, IQR:2–3) (Figure 2). Medical students also agreed that colleagues speak positively of LGB individuals (median = 2, IQR:1–2), and less positively of transgender individuals (median = 3, IQR:2–3). They overall agreed that medical students are supportive of LGBT colleagues if they are treated negatively (median = 2, IQR:1–3). Students were not of the opinion that LGBT colleagues are subjected to negative comments and/or jokes (median = 4, IQR:2–4), rumors (median = 4, IQR:3–5), or bullying and/or harassment (median = 4, IQR:4–5).

In comparison to cis-gender heterosexual, LGBT students’ opinions were less positive (Figure 2). They expressed a less fair treatment of transgender students (p < 0.001), and a lower agreement with medical students speaking positively of LGB (p = 0.04) or transgender individuals (p = 0.008). Finally, they agreed more strongly with the statements that LGBT colleagues are subjected to negative comments and/or jokes (p < 0.001), rumors (p = 0.02), or bullying and/or harassment (p = 0.006). Overall, transgender issues were more pronounced than those of sexual orientation minorities. Students indicated that transgender students were treated less fairly in comparison to LGB students (median = −1, IQR:-2–0, p < 0.001), and that transgender individuals were spoken of in a less positive manner (median = 0, IQR:-1–0, p < 0.001).

Students were also asked about LGBT related professional activities (volunteer, research, and/or advocacy) that they were involved in. They agreed with the statement that they were comfortable disclosing such activities during their residency applications (median = 2, IQR:1–3). However, LGBT students felt less comfortable disclosing this information when compared to cis-gender heterosexual ones (p = 0.007).

### Awareness

An overwhelming majority of medical students felt confident in defining the terms gay (99.0%, n = 102/103), lesbian (98.1%, n = 101), homosexual (98.1%, n = 101), sexual orientation (97.1%, n = 100), homophobia (97.1%, n = 100), bisexual (96.1%, n = 99), and transgender (91.3%, n = 94) (Table 3). Respondents were less confident with the terms LGBT (66.0%, n = 68) and queer (46.6%, n = 48), and only 21.4% (n = 22) were able to define two spirited. LGBT students had higher odds being confident in defining LGBT (OR = 4.0, 95%CI:1.39–13.3, p = 0.005), queer (OR = 3.89, 95%CI:1.53–10.1, p = 0.002), and two-spirited (OR = 22.2, 95%CI:5.4–125, p < 0.001). The majority of survey respondents felt comfortable providing medical care to patients regardless of their gender identity and/or sexual orientation. This was the case for both cis-gender heterosexual and LGBT students.

**Table 3.** Self-reported knowledge of LGBT terminology and comfort level providing medical care to members of the LGBT community.

	Number	Percentage (%)
**Term definitions**		
Gay	102	99.0
Lesbian	101	98.1
Homosexual	101	98.1
Sexual orientation	100	97.1
Homophobia	100	97.1
Bisexual	99	96.1
Transgender	94	91.3
Gender identity	87	84.5
LGBT	68	66.0
Queer	48	46.6
Two spirited	22	21.4
		
**Medical care**		
Gay	100	97.1
Lesbian	100	97.1
Bisexual	99	96.1
Queer	89	86.4
Transgender	86	83.5
Two spirited	79	76.7
		

LGBT: Lesbian, gay, bisexual, or transgender individuals. Two spirited: A term used by North American indigenous LGBT people.

In regards to sexual orientation, the majority of students believed individuals are born this way (79.6%, n = 82) (Table 4). Around a third stated that they are not sure or that it is difficult to explain (34.0%, n = 35). Non-LGBT students had higher odds selecting not sure (OR = 3.16, 95%CI:1.13–9.7, p = 0.018) and lower odds in choosing being born this way (OR = 0.064, 95%CI:0.0015–0.45, p < 0.001). As for transgender individuals, a similar pattern is noted. Non-LGBT students had higher odds selecting not sure (OR = 3.80, 95%CI:1.36–11.6, p = 0.006), and lower odds selecting being born this way (OR = 0.189, 95%CI:0.0339–0.72, p = 0.007).

**Table 4.** Personal beliefs on why individuals are LGBT.

	LGB		Transgender	
Reason for being	Number^a^	Percentage (%)	Number^a^	Percentage (%)
Born this way	82	79.6	79	76.7
Not sure	35	34.0	38	36.9
Personal choice	18	17.5	14	13.6
Upbringing	18	17.5	12	11.7
Other	5	4.9	5	4.9

^a^Numbers do not add up to total number of surveys as respondents were allowed to select multiple choices.

LGB: Lesbian, gay, and/or bisexual individuals. LGBT: Lesbian, gay, bisexual, or transgender individuals.

### Safe space promotion and LGBT education

Students were asked to select which measures they believe should be taken to promote equality and create a safe space (Online, Appendix 3). The majority agreed with offering additional training/education around LGBT issues (i.e., sexual orientation, gender identity, inclusiveness, respect) (75.7%, n = 78). Overall, students expressed a high interest in obtaining further education around LGBT issues (84.5%, n = 87), and workshops was the most commonly chosen format to receive such information (70.9%, n = 73). LGBT students had higher odds of supporting additional training around LGBT issues (OR = 46, 95%CI:2.71–784, p < 0.001) and services specific to LGBT students (OR = 2.74, 95%CI:1.11–6.8, p = 0.02).

## Discussion

This survey study sought to assess levels of heterosexism and anti-LGBT discrimination experienced by medical students at the University of Ottawa and their comfort level in providing care to patients of gender and sexual minorities. In the past decades, acceptance of sexual and gender minorities in western societies has continued to rise [8]. This has translated into significant gains for these minorities, from anti-discrimination laws to marriage equality in more than twenty countries so far. Our results reflected this greater level of acceptance. Students felt comfortable providing medical care to LGBT patients and were open for further training. They believed that LGBT individuals are born that way (79.6%); much higher than the general population as a recent American poll suggests (41%) [30]. Further, these results demonstrate progress relative to a similar survey conducted in 2013 with staff, physicians, and trainees at a teaching hospital of the University of Ottawa. Compared to the respondents of four years ago, the students surveyed here were more confident with their ability to define LGBT terminology, more confident of their ability to provide quality care to LGBT patients, and among LGBT respondents, were more likely to disclose their sexual orientation [24]. Understanding LGBT terminology is a the first step in providing a more welcoming and inclusive environment for LGBT patients.

Similar to what was noted previously with visible minorities, with advancement in LGBT rights, explicit bias and discrimination tend to drop, as they are usually susceptible to a social desirability bias. However, implicit bias requires a longer period before a shift is noted [10,11]. Our results followed a similar pattern, where overt discrimination against LGBT individuals was low in prevalence, while measures of the inclusiveness of the environment showed that there is room for improvement. The most common source of heterosexism and/or LGBT discrimination were fellow medical students which was more than twice as common as the next largest group; attending or staff physicians in either a clinical or academic setting. Nonetheless, our results were more positive than previously published studies, where a quarter of LGBT physicians were socially ostracized [14], and a half of general surgery residents had witnessed homophobic remarks by colleagues or staff physicians [31]. This difference between our study and previous literature can be explained by multiple reasons, such as overall societal shift in terms of LGBT rights, as well as cultural and national differences. Whereas our study is Canadian, most previous studies have been completed in the USA.

A recent study found LGB medical students to be more likely to report social stressors (including harassment) as well as social isolation [1], placing them at greater risk of depression and anxiety. In our study, LGBT students had higher odds for witnessing overt discrimination and for describing a less inclusive environment. All our LGBT survey respondents have shared their gender and/or sexual minority status with some of their colleagues, and none concealed it completely (Figure 1). However, students tended not to share this with their supervisors in a classroom or a clinical setting (ex: Residents, allied health members and staff physicians). Similarly, LGBT students were less likely to feel comfortable sharing LGBT related activities on their residency application, but our results remain more promising than previous literature. In a large study of all undergraduate medical training programs in the USA and Canada, it was found that almost one third of LGBT medical students conceal their sexual identity in medical school [32]. Similar to our findings, students there listed numerous reasons that included perceived lack of a supportive environment; fear of discrimination from peers and/or faculty; and fear of exclusion from future career options [33].

Issues of gender identity continue to be a source of societal debate, and our results followed that trend. While the overall opinion of treatment and acceptance of LGB students and individuals was mostly favorable among medical students, transgender issues tended to be less optimal (Figure 2). Medical students felt less comfortable treating transgender patients, and students stated the lack of knowledge and training on transgender specific health issues as reasons for this. Prior publications demonstrate a similar pattern beyond the undergraduate level. Among adolescent health care providers, only 47.1% felt confident providing adequate medical therapy to transgender youth, with 65.1% indicating that lack of training as a major barrier [34, 35]. Identifying these gaps by healthcare providers is the first step in improving the curriculum. Similarly, more than two thirds of graduating medical students have described their LGBT curriculum as fair or worse, and only a minority (26.1–28.0%) felt comfortable in regards to transgender health issues [36]. However, most students in our study reported desire for further education around LGBT health issues (84.5%). Recent studies show that medical students are willing to participate in LGBT related training when offered the opportunity, even if it entailed a significant time commitment [37].

This study provides a significant contribution to the field of LGBT healthcare and related medical education. It is the first study to target specifically a Canadian environment. Strengths of this study include the assessment of overt discrimination and the overall inclusiveness of the school environment to LGBT medical students. As well, it was able to assess medical students’ acceptance towards LGBT patients and their comfort level in providing care to sexual and gender minorities. Finally, the study was able to provide a comparison between cis-gender heterosexual and LGBT studentsʼ views and perceptions on these issues. Despite these strengths, the study has some limitations with a response rate at 15.4%. However, this is comparable to other studies in the field, where study participation was optional and did not offer any incentive [15]. As well, the study targeted only one medical school, and a broader study is required to be able to assess Canadian medical schools overall. Finally and similar to literature, LGBT students represented a high proportion among respondents (35.9%), likely higher than the true proportion among medical students estimated at 5.0% [1]. The higher response rate noted in our study can be explained by LGBT students showing a higher interest in healthcare issues related to their own community [35]. This disproportionate response does not allow us to generalize our results. Similarly, heterosexual allies who view LGBT issues as important may also have been more likely to respond to the survey. This can lead to an overestimation of the prevalence of LGBT discrimination and heterosexism, as well as the comfort level in treating LGBT patients, and understanding of LGBT related terms.

While the body of literature covering disparities with regard to LGBT health, in both healthcare and medical education is growing, multiple gaps remain to be addressed. Research has been focused on demonstrating these inequalities, and the prevalence of discrimination in the healthcare field. However, there is a lack of evidence on the best approach to change this. Future studies should assess strategies to improve on the schools’ inclusiveness to sexual and gender minorities, and to target deficits in the curriculum.

## Conclusions

The overall results show low explicit bias, with students feeling comfortable providing care to most of the LGBT community. There was a consensus among students that further education and training regarding LGBT issues is needed as well as desired. However, incidences of heterosexism and to a lesser degree discrimination against LGBT individuals continue to occur. These were more perceived and/or witnessed by LGBT medical students in comparison to cis-gender heterosexual students. These factors and the fear of losing professional opportunities continue to weigh on LGBT medical students and impact their learning experience.

## Supplementary Material

Appendices.zip
