# Supplementary material for: Medical students’ perception of lesbian, gay, bisexual, and transgender (LGBT) discrimination in their learning environment and their self-reported comfort level for caring for LGBT patients: a survey study
Source: Med Educ Online. 2017 Aug 30;22(1):1368850. doi: 10.1080/10872981.2017.1368850 (PMC5653936; doi:10.1080/10872981.2017.1368850)
Supplement: Appendices.zip [file ZMEO_A_1368850_SM4665.zip › Appendix 1.pdf]

**Medical Students' Attitude Around Lesbian, Gay, Bisexual, and Transgender (LGBT) Issues for Patients and Colleagues: A Survey Study**

Nama et al. Medical Education Online. 2017

**Thank you for completing this confidential and anonymous survey.**
**If you feel uncomfortable answering any question(s), you may choose to leave the question(s) unanswered and/or stop this survey at any time.**

## A. BACKGROUND

1. What is your current year of study?
   a. First
   b. Second
   c. Third
   d. Fourth
   e. Prefer not to say

2. Prior to beginning the MD program what was the highest level of academic training you had obtained?
   a. Bachelor degree (e.g. BSc/BScH, BA/BAH, etc) was started but not completed
   b. Bachelor degree (e.g. BSc/BScH, BA/BAH, etc) was completed
   c. Master degree (e.g. MSc, MA) was completed
   d. Doctorate degree (e.g. PhD) was completed

## B. GENDER AND SEXUAL ORIENTATION

1. What gender do you identify as?
   a. Male
   b. Female
   c. Transgender
   d. Two Spirited
   e. Not sure
   f. Other [if selected, free text box will open – "Please describe: ____________"]
   g. Prefer not to answer

2. What is your sexual orientation?
   a. Heterosexual [IF 'HETEROSEXUAL' IS CHOSEN, SKIP TO DISCRIMINATION SECTION]
   b. Bisexual
   c. Gay
   d. Lesbian
   e. Queer
   f. Questioning
   g. Not sure
   h. Other [if selected, free text box will open – "Please describe: ____________"]
   i. Prefer not to answer

3. Please list to whom in your class are you "open" to regarding your sexual orientation and/or gender identity? (check the most appropriate answer)
   a. No one
   b. Some students or classmates in your year
   c. All students or classmates in your year
   d. All students in your program

4. Please list who you are "open" to regarding your sexual orientation and/or gender identify? (check all that apply):

| | | | | |
|---|---|---|---|---|
| a. No one | | | | |
| b. Staff members at the UGME office | None? | Some? | All? | N/a? |
| c. Allied health members | None? | Some? | All? | N/a? |
| d. Residents | None? | Some? | All? | N/a? |
| e. Staff Physician Tutors (i.e. CBL, PSD) | None? | Some? | All? | N/a? |
| f. Staff Physician Clinical Supervisors | None? | Some? | All? | N/a? |

5. If you are not "fully" open at work about your sexual orientation and/or gender identity, please explain your reasons: (check all answers that apply)
   a. Concern about what other medical students may think / say
   b. Concern regarding your personal safety
   c. Concern that you may be discriminated against (i.e., lost career opportunities, negative impact upon evaluations)
   d. Concern that it may affect your choice of residency options in CaRMS
   e. Concern that you may be stereotyped
   f. Prior personal experience with harassment / discrimination regarding LGBTQ issues
   g. Prior experience of others being subjected to harassment or discrimination regarding LGBTQ issues
   h. Personal belief that this information is not anyone else's business
   i. Other: [Please specify]:_________________________________

6. If you were active in LGBTQ volunteer activities, research and/or advocacy, how comfortable would you feel listing this on your CaRMS application when applying for a Residency position?
   a. Very likely
   b. Likely
   c. Neutral
   d. Unlikely
   e. Very unlikely

## C. DISCRIMINATION

1. Have you witnessed and/or have you been exposed to heterosexism by someone in or affiliated with the University of Ottawa medical school program? (**Heterosexism** is defined as favouring opposite-sex sexuality and relationships and/or portraying opposite-sex relationships as the only norm and therefore superior)
   a. Yes
   b. No [IF 'NO' IS CHOSEN, SKIP TO #3 IN THIS SECTION]

2. Please list the individual(s) that were the source of the heterosexism (check all that apply):
   a. Colleagues (i.e. other medical students) in my own year
   b. Colleagues (i.e. other medical students in other years
   c. Staff members at the UGME office
   d. Residents
   e. Staff Physician Tutors (i.e. PBL, PSD)
   f. Staff Physician Clinical Supervisors
   g. Nurses
   h. Operating room staff
   i. Physiotherapists
   j. Occupational therapists
   k. Other [Please give examples: ____________________________]

3. Have you witnessed and/or have been exposed to discrimination against an LGBTQ individual by someone in or affiliated with the University of Ottawa medical school program?
   a. Yes
   b. No [IF 'NO' IS CHOSEN, SKIP TO GENERAL DISCRIMINATION SECTION]

4. Please list the individual(s) that were the source of the discrimination against LGBTQ individual (check all that apply):
   a. Colleagues (i.e. other medical students) in my own year
   b. Colleagues (i.e. other medical students in other years
   c. Staff members at the UGME office
   d. Residents
   e. Staff Physician Tutors (i.e. PBL, PSD)
   f. Staff Physician Clinical Supervisors
   g. Nurses
   h. Operating room staff
   i. Physiotherapists
   j. Occupational therapists
   k. Other [Please give examples: ____________________________]

5. Please provide non-identifying examples if you feel comfortable sharing [FREE TEXT BOX]

6. Do you feel that the discrimination that you witnessed made you or another individual feel of less value and/or less self-worth?
    a. Yes
    b. No

7. Do you feel that the discrimination that you witnessed made you or another individual feel of physically threatened or at risk?
    a. Yes
    b. No

## D. GENERAL DISCRIMINATION

1. Lesbian, gay, and bisexual students are treated fairly in your program of study
    a. Strongly agree
    b. Agree
    c. Neutral
    d. Disagree
    e. Strongly agree

2. Transgender students are treated fairly in your program of study
    a. Strongly agree
    b. Agree
    c. Neutral
    d. Disagree
    e. Strongly agree

**How often each of the following happen in your program of study? Please check the box that best describes your experience.**

3. Other medical students at uOttawa speak of lesbian, gay and/or bisexual persons in a positive way
    a. Strongly agree
    b. Agree
    c. Neutral
    d. Disagree
    e. Strongly agree

4. Other medical students at uOttawa speak of transgender persons in a positive way
    a. Strongly agree
    b. Agree
    c. Neutral
    d. Disagree
    e. Strongly agree

5. Other medical students at uOttawa speak up and/or show support for a LGBTQ colleague if they are treated negatively
    a. Strongly agree
    b. Agree
    c. Neutral
    d. Disagree
    e. Strongly agree

6. Negative comments and/or jokes are told that may be hurtful to LGBTQ persons
    a. Strongly agree
    b. Agree
    c. Neutral
    d. Disagree
    e. Strongly agree

7. Rumours are spread about your own or someone else's sexual orientation and/or gender identity
    a. Strongly agree
    b. Agree
    c. Neutral
    d. Disagree
    e. Strongly agree

8. Bullying, harassment, and/ or discrimination against LGBTQ students takes place
    a. Strongly agree
    b. Agree
    c. Neutral
    d. Disagree
    e. Strongly agree

## E. AWARNESS

1. Please indicate which terms below you feel you could accurately define or explain. (Check all answers that apply).
    a. Gay
    b. Homophobia
    c. Gender Identity
    d. Homosexual
    e. Lesbian
    f. Sexual Orientation
    g. Bisexual
    h. Transgender
    i. Two Spirited
    j. GLBTQ
    k. Queer
    l. Not sure that I can accurately define or explain any of the terms

2. I would feel comfortable providing medical care for persons who self-identify as:

| | | |
|---|---|---|
| a. Lesbian | Yes | No |
| b. Gay | Yes | No |
| c. Bisexual | Yes | No |
| d. Transgender | Yes | No |
| e. Two Spirited | Yes | No |
| f. Queer | Yes | No |

3. Please explain your belief as to why people are lesbian, gay, or bisexual? Please check all that apply.
   a. They are born that way
   b. It is due to their upbringing
   c. It is a personal choice
   d. Not sure/Difficult to explain
   e. Other: [Please explain]:___________________________________

4. Please explain your belief as to why people are transgendered? Please check all that apply.
   a. They are born that way
   b. It is due to their upbringing
   c. It is a personal choice
   d. Not sure/Difficult to explain
   e. Other: [Please explain]:___________________________________

5. Comments: Please explain or clarify responses for this section, if you'd like. [FREE TEXT BOX]

## F. EFFORTS TO PROMOTE EQUALITY AND CREATE A SAFE SPACE

1. What are important steps that your program could take to create a more accepting environment for GLBTQ students? (Check all answers that apply)
   a. Offer additional training/ education around GLBTQ issues (i.e., sexual orientation, gender identity, inclusiveness, respect)
   b. Promote the usage of GLBTQ friendly language and images
   c. Make public the steps that your program is taking to make the school a more GLBTQ friendly space
   d. Offer services specific to GLBTQ students (i.e. a student counselor to deal with GLBTQ concerns and complaints)
   e. Gender neutral washrooms
   f. Unsure/ Difficult to explain
   g. Other (please specify below)
   h. If you answered "other", please specify:___________________________________

2. Would you be interested in education around LGBTQ issues?
    a. Yes
    b. No

3. If yes, what would you like to learn more about?
    a. ________________________________

4. If yes, how would you like to receive information?
    a. Written materials
    b. Workshops
    c. Videos
    d. Other
    e. If you answered "other", please specify:________________________________

**Nous vous remercions d'avoir rempli ce sondage confidentiel et anonyme.**

## A. ARRIÈRE-PLAN

1. Quelle est votre année d'études ?
   a. Première
   b. Deuxième
   c. Troisième
   d. Quatrième
   e. Je préfère ne pas répondre

2. Avant le début du programme MD ce qui était le plus haut niveau de formation académique que vous aviez obtenue ?
   a. Diplôme de Baccalauréat (ex : BSc / BScH, BA/BAH, etc.) a été commencé, mais pas terminé
   b. Diplôme de Baccalauréat (ex : BSc / BScH, BA/BAH, etc.) a été complété
   c. Diplôme de Maîtrise (ex : M.Sc., MA) a été complété
   d. Diplôme de Doctorat (ex : PhD) a été complété

## B. GENRE ET ORIENTATION SEXUELLE

1. Quel est le sexe dont vous identifiez ?
   a. Mâle
   b. Femelle
   c. Transgenre
   d. Bispiritualité
   e. Pas sûr
   f. Autres [si ceci est sélectionné, une zone de texte libre ouvrira — « S'il vous plaît, décrire : ________ »]
   g. Je préfère ne pas répondre

2. Quelle est votre orientation sexuelle ?
   a. Hétérosexuel [SI CECI EST CHOISI, PASSER À LA SECTION SUR LA DISCRIMINATION]
   b. Bisexuel
   c. Gai
   d. Lesbienne
   e. Queer
   f. En questionnement
   g. Pas sûr
   h. Autres [si ceci est sélectionné, une zone de texte libre ouvrira — « S'il vous plaît, décrire : ________ »]
   i. Je préfère ne pas répondre

3. S'il vous plaît, énumérer avec qui dans votre classe vous êtes à l'aise pour ce qui concerne votre orientation sexuelle et/ou votre identité de genre. (Choisissez la réponse la plus appropriée)
    a. Personne
    b. Certains étudiants ou camarades de classe dans votre année
    c. Tous les étudiants ou camarades de classe dans votre année
    d. Tous les étudiants de votre programme

4. S'il vous plaît, énumérer avec qui vous êtes à l'aise pour ce qui concerne votre orientation sexuelle et/ou votre identité de genre. (Choisissez toutes les réponses appropriées)
    a. Personne
    b. Membres du personnel du bureau ÉMPC Aucun ? Certains ? Tout ? N/a ?
    c. Autres professionnels de santé Aucun ? Certains ? Tout ? N/a ?
    d. Des résidents Aucun ? Certains ? Tout ? N/a ?
    e. Des médecins tuteurs (APC, DAC) Aucun ? Certains ? Tout ? N/a ?
    f. Des médecins superviseurs clinique Aucun ? Certains ? Tout ? N/a ?

5. Si vous n'êtes pas « complètement » ouvert au travail pour ce qui concerne votre orientation sexuelle et/ou votre identité de genre, s'il vous plaît expliquer vos raisons : (Choisissez toutes les réponses appropriées)
    a. Préoccupation à propos de ce que les autres étudiants en médecine peuvent penser/dire
    b. Préoccupation concernant votre sécurité personnelle
    c. Crainte que vous puissiez être victime de discrimination (ex. : perdre des possibilités de carrière, l'impact négatif sur les évaluations)
    d. Crainte que cela puisse influer sur votre choix de résidence à CaRMS
    e. Crainte que vous puissiez être stéréotypé
    f. Une expérience personnelle préalable avec du harcèlement/de la discrimination en ce qui concerne les questions LGBTQ
    g. Une expérience préalable où des autres ont été soumis au harcèlement ou à la discrimination en ce qui concerne les questions LGBTQ
    h. Croyance personnelle que cette information ne concerne pas les autres.
    i. Autre (veuillez préciser) : ___________________________________

6. Si vous étiez actif dans des activités LGBTQ bénévolat, recherche et/ou de support, vous sentiriez-vous confortable de les énumérer dans votre application de CaRMS lors de vos demandes de résidence ?
    a. Très probable
    b. Probable
    c. Neutre
    d. Improbable
    e. Très improbable

## C. DISCRIMINATION

1. Avez-vous été témoin et/ou avez-vous été exposé à l'hétérosexisme par quelqu'un affilié au programme de médecine de l'Université d'Ottawa ? (**Hétérosexisme** est défini comme le favorisent envers la sexualité et les relations entre sexes opposés et/ou la description des relations entre sexes opposés comme la seule norme et donc supérieures).
   a. Oui
   b. Non [si « Non » est choisi, passer à la question #3 dans cette section]

2. S'il vous plaît, énumérer l'individu(e) qui a été la source de l'hétérosexisme (cochez toutes les cases applicables) :
   a. Collègues (ex. d'autres étudiants en médecine dans ma propre année)
   b. Collègues (ex. autres étudiants en médecine dans d'autres années)
   c. Membres du personnel du bureau ÉMPC
   d. Résidents
   e. Médecins Tuteurs (ex. APC, DAC)
   f. Médecin Superviseurs Cliniques
   g. Infirmières
   h. Personnel de la salle d'opération
   i. Kinésithérapeutes
   j. Ergothérapeutes
   k. Autres [s'il vous plaît, donnez des exemples : ____________________________]

3. Avez-vous été témoin et/ou été exposé à la discrimination contre une personne LGBTQ par quelqu'un affilié au programme de médecine de l'Université d'Ottawa ?
   a. Oui
   b. Non [si « Non » est choisi, passer à la section de discrimination générale]

4. S'il vous plaît, énumérer l'individu (e) qui a été la source de la discrimination à l'égard des individus LGBTQ (cochez toutes les cases applicables) :
   a. Collègues (ex. d'autres étudiants en médecine dans ma propre année)
   b. Collègues (ex. autres étudiants en médecine dans d'autres années)
   c. Membres du personnel du bureau ÉMPC
   d. Résidents
   e. Médecins Tuteurs (ex. APC, DAC)
   f. Médecin Superviseurs Cliniques
   g. Infirmières
   h. Personnel de la salle d'opération
   i. Kinésithérapeutes
   j. Ergothérapeutes
   k. Autres [s'il vous plaît, donnez des exemples : ____________________________]

5. S'il vous plaît, fournir des exemples non identifiables si vous vous sentez à l'aise de les partager [ZONE DE TEXTE LIBRE]

6. Pensez-vous que la discrimination dont vous avez été témoin vous a fait sentir ou a fait sentir quelqu'un d'autre moins de valeur et/ou moins d'estime de soi ?
    a. Oui
    b. Non

7. Pensez-vous que la discrimination dont vous avez été témoin vous a fait sentir ou a fait sentir quelqu'un d'autre en menace physique ou en danger ?
    a. Oui
    b. Non

## D. DISCRIMINATION GÉNÉRALE

1. Les étudiant(e)s lesbiennes, gaies et bisexuelles sont traitées équitablement dans votre programme d'études
    a. Tout à fait d'accord
    b. D'accord
    c. Neutre
    d. Désaccord
    e. Totalement en désaccord

2. Les étudiant(e)s transgenres sont traités équitablement dans votre programme d'études
    a. Tout à fait d'accord
    b. D'accord
    c. Neutre
    d. Désaccord
    e. Totalement en désaccord

**Combien de fois chacun des éléments suivants se produit dans votre programme d'études ? S'il vous plaît, cochez la case qui décrit le mieux votre expérience.**

1. Des étudiant(e)s en médecine à l'Université d'Ottawa parlent des personnes bisexuelles lesbiennes, et/ou gaies d'une manière positive
    a. Tout à fait d'accord
    b. D'accord
    c. Neutre
    d. Désaccord
    e. Totalement en désaccord

2. Des étudiants en médecine à l'Université d'Ottawa parlent des personnes transgenres d'une manière positive
    a. Tout à fait d'accord
    b. D'accord
    c. Neutre
    d. Désaccord
    e. Totalement en désaccord

3. Des étudiants en médecine à l'Université d'Ottawa parlent et/ou montrent du soutien pour un collègue LGBTQ s'ils sont traités négativement
    a. Tout à fait d'accord
    b. D'accord
    c. Neutre
    d. Désaccord
    e. Totalement en désaccord

4. Des commentaires et/ou des blagues négatives sont dits qui peuvent être blessants pour les personnes LGBTQ
    a. Tout à fait d'accord
    b. D'accord
    c. Neutre
    d. Désaccord
    e. Totalement en désaccord

5. Des rumeurs se propagent à propos de votre propre orientation sexuelle et/ou identité de genre ou celles d'un autre
    a. Tout à fait d'accord
    b. D'accord
    c. Neutre
    d. Désaccord
    e. Totalement en désaccord

6. De l'intimidation, du harcèlement, et/ou de la discrimination ont lieu contre les étudiants LGBTQ
    a. Tout à fait d'accord
    b. D'accord
    c. Neutre
    d. Désaccord
    e. Totalement en désaccord

## E. SENSIBILISATION

1. S'il vous plaît, indiquez parmi les termes ci-dessous lesquelles vous sentez que vous pouvez définir ou expliquer précisément. (Cochez toutes les réponses applicables).
    a. Gai
    b. Homophobie
    c. Identité de genre
    d. Homosexuel
    e. Lesbienne
    f. Orientation sexuelle
    g. Bisexuel
    h. Transgenre
    i. Bispiritualité
    j. GLBTQ
    k. Queer
    l. Pas sûr que je peux définir ou expliquer précisément l'un de ces termes

2. Je me sentirais à l'aise de fournir des soins médicaux pour les personnes qui s'identifient comme :

| | | |
|---|---|---|
| a. Lesbienne | Oui | Non |
| b. Gai | Oui | Non |
| c. Bisexuel | Oui | Non |
| d. Transgenre | Oui | Non |
| e. Bispiritualité | Oui | Non |
| f. Queer | Oui | Non |

3. S'il vous plaît expliquer votre croyance de pourquoi des gens sont gais, lesbiennes, ou bisexuels ? S'il vous plaît, cochez toutes les cases applicables.

    a. Ils sont nés de cette façon
    b. C'est dû à leur éducation
    c. C'est un choix personnel
    d. Pas sûr/difficile à expliquer
    e. Autre : [s'il vous plaît, expliquer] : ___________________________________

4. S'il vous plaît expliquer votre croyance de pourquoi des gens sont transgenres ? S'il vous plaît, cochez toutes les cases applicables.

    a. Ils sont nés de cette façon
    b. C'est dû à leur éducation
    c. C'est un choix personnel
    d. Pas sûr/difficile à expliquer
    e. Autre : [s'il vous plaît, expliquer] : ___________________________________

5. Commentaires : S'il vous plaît, expliquer ou clarifier les réponses de cette section, si vous le souhaitez. [ZONE DE TEXTE LIBRE]

## F. EFFORTS POUR PROMOUVOIR L'ÉGALITÉ ET CRÉER UN ESPACE SUR

1. Quelles sont les étapes importantes que votre programme pourrait prendre pour créer un environnement plus accueillant pour les étudiants GLBTQ? (Cochez toutes les réponses applicables).
    a. Offrir une formation supplémentaire autour des questions de GLBTQ (ex. l'orientation sexuelle, l'identité de genre, l'inclusion, le respect)
    b. Promouvoir l'utilisation d'un langage et des images plus conviviales aux GLBTQ
    c. Rendre publiques les mesures que votre programme prend pour faire de l'école un espace plus convivial aux GLBTQ.
    d. Offrir des services spécifiques aux étudiants GLBTQ (ex. un conseiller pour faire face à des préoccupations et des plaintes GLBTQ)
    e. Toilettes de genre neutre
    f. Pas sûr/difficile à expliquer
    g. Autre (s'il vous plaît, précisez ci-dessous)
    h. Si vous avez répondu « autre », s'il vous plaît préciser : ____________________

2. Seriez-vous intéressé à une éducation sur les questions LGBTQ ?
    a. Oui
    b. Non

3. Si oui, que voulez-vous en savoir plus sur ?
    a. ___________________________________

4. Si oui, comment voulez-vous recevoir de l'information ?
    a. Des documents écrits
    b. Ateliers
    c. Vidéos
    d. Autre
    e. Si vous avez répondu « autre », s'il vous plaît préciser : ____________________
