# Supplementary figures and images for: Medical students’ perception of lesbian, gay, bisexual, and transgender (LGBT) discrimination in their learning environment and their self-reported comfort level for caring for LGBT patients: a survey study
Source: Med Educ Online. 2017 Aug 30;22(1):1368850. doi: 10.1080/10872981.2017.1368850 (PMC5653936; doi:10.1080/10872981.2017.1368850)

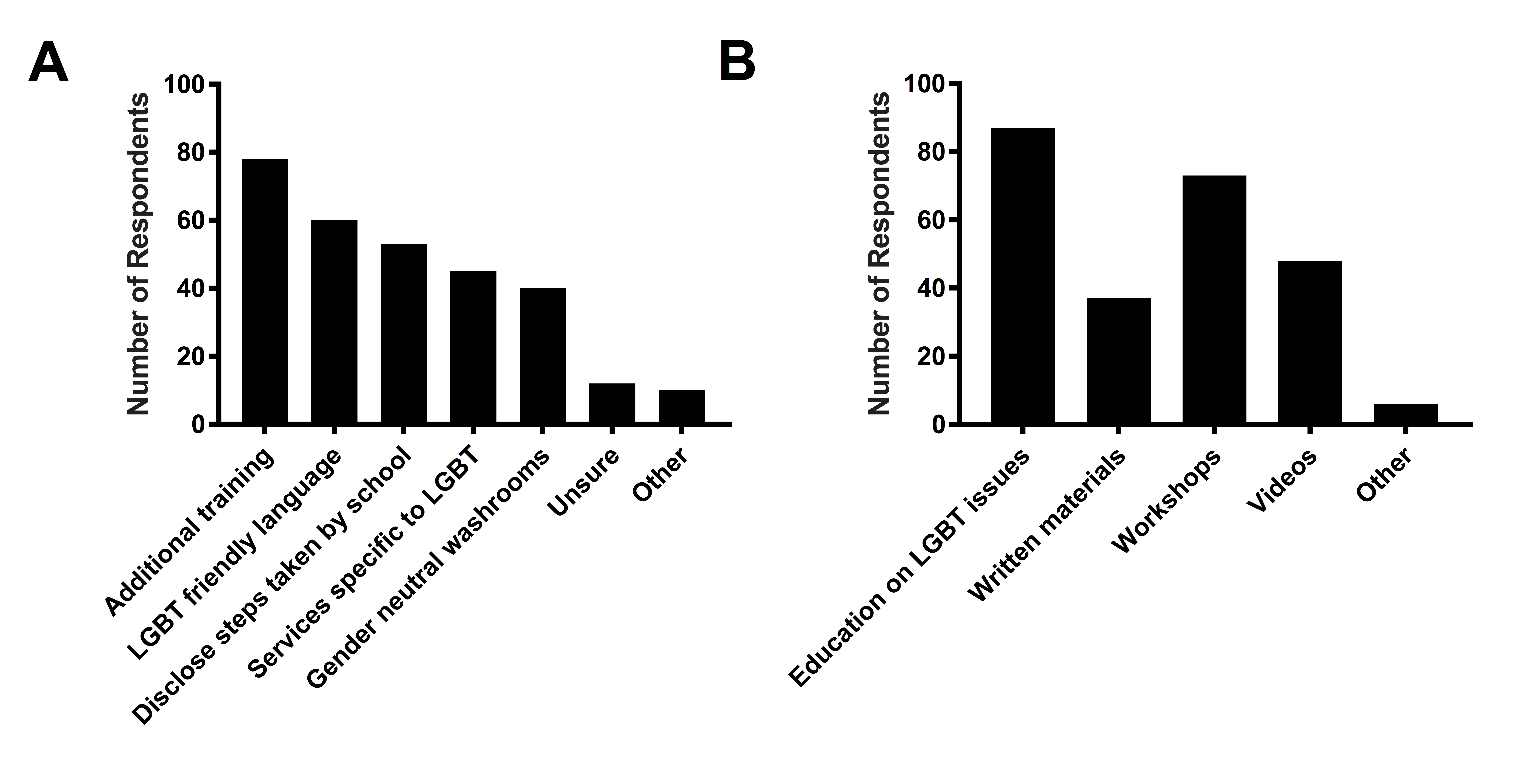

Supplement: Appendices.zip [file ZMEO_A_1368850_SM4665.zip › Appendix 3.jpg]

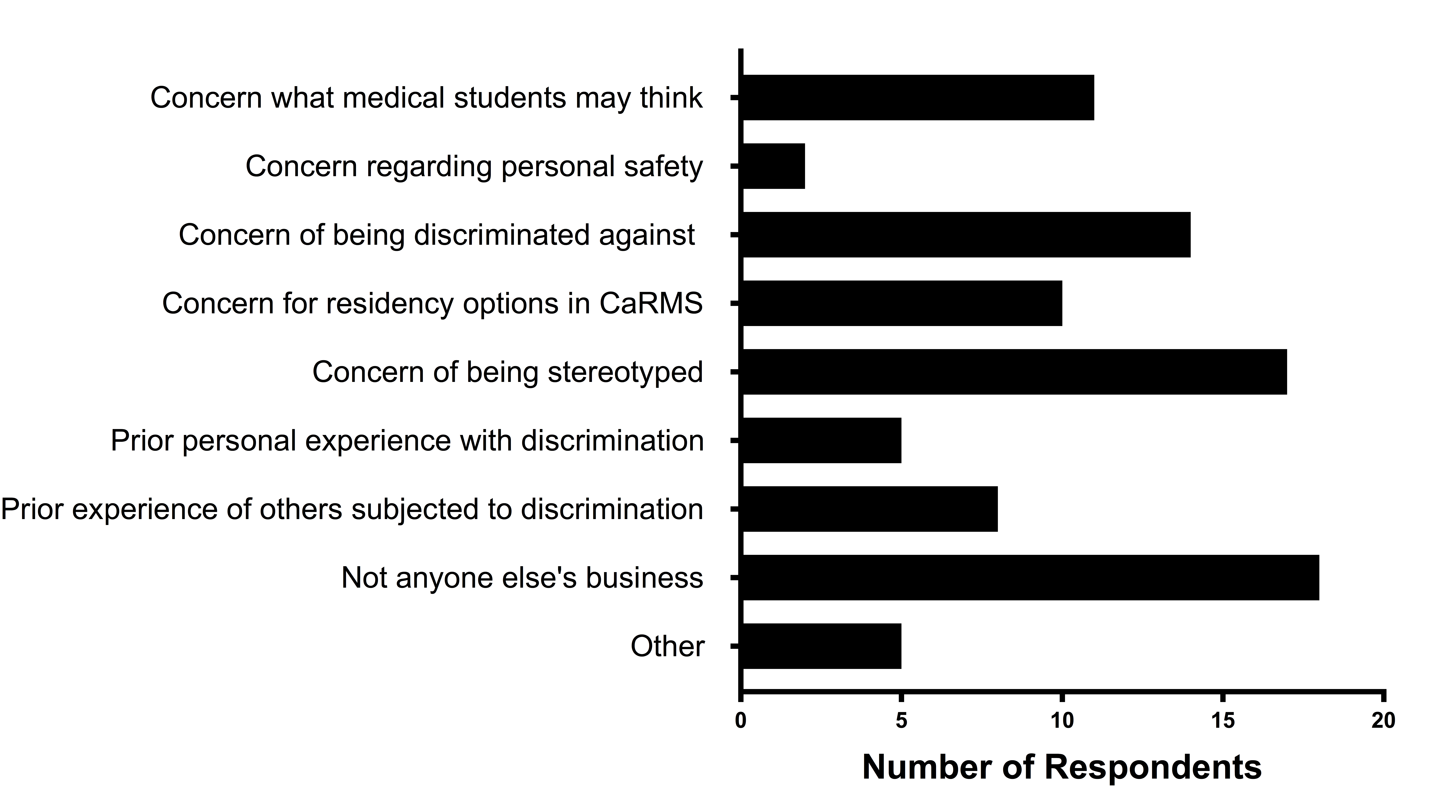

Supplement: Appendices.zip [file ZMEO_A_1368850_SM4665.zip › Appendix 2.jpg]
